# Structural Basis for the Immunological Paradox of a High-Affinity Yet Non-Immunogenic MHC-I Epitope from *Cryptosporidium parvum*

**DOI:** 10.3390/biology15151281

**Published:** 2026-08-04

**Authors:** Shuhua Fan, Tingting Wang, Shuaihao Ren, Jiajia Peng, Luqi Li, Yuanyuan Zhao, Jiaqi Yang, Yizhou Zhang, Yaqun Yan, Hongxing Wang, Yongli Wang

**Affiliations:** 1 College of Life Sciences and Agronomy, Zhoukou Normal University, Zhoukou 466001, China; 20011025@zknu.edu.cn (S.F.); 202307010011@zknu.edu.cn (T.W.); 202307050010@zknu.edu.cn (S.R.); 202307010057@zknu.edu.cn (J.P.); 202407010017@zknu.edu.cn (L.L.); 202407050042@zknu.edu.cn (Y.Z.); 202507140037@zknu.edu.cn (J.Y.); 202507050039@zknu.edu.cn (Y.Z.); yanyq2022@zknu.edu.cn (Y.Y.); 2 Fuxi Laboratory, Zhoukou 466001, China; 3 State Key Laboratory of Agricultural Microbiology, College of Veterinary Medicine, Huazhong Agricultural University, Wuhan 430070, China

**Keywords:** MHC I, *Cryptosporidium parvum*, peptide epitope, structural biology, immune evasion

## Abstract

*Cryptosporidium parvum* is an important apicomplexan parasite that causes severe diarrheal disease in children and immunocompromised individuals. However, the structural basis for the limited immunogenicity of its T-cell epitopes remains poorly understood. This study integrates structural biology and immunological approaches to elucidate the molecular basis underlying the non-immunogenicity of KAV9, a Cp23-derived epitope with the sequence KAVKNPAPI. Biophysical analyses demonstrated that KAV9 forms a high-affinity complex with H-2D^b^, with an IC_50_ of 7.83 nM, and exhibits higher thermal stability (*T*_m_ = 59.16 °C) than the immunodominant LCMV gp33 epitope (*T*_m_ = 51.15 °C). Despite strong pMHC binding and high pMHC stability, in vivo peptide immunization failed to elicit a detectable KAV9-specific CD8^+^ T-cell response. Crystal structure analysis revealed that KAV9 is tightly accommodated within the H-2D^b^ binding groove through an extensive hydrogen-bond network. However, its distinct peptide conformation, particularly involving P4-Lys and the proline residues at P6 and P8, markedly reshapes the TCR-exposed surface compared to gp33. AlphaFold3 (AF3) modeling further suggested that these structural deviations disrupt critical hydrogen-bond interactions with the T-cell receptor (TCR) CDR3 loops, thereby eliminating contacts required for TCRβ engagement. Sequence analysis revealed that KAV9 is highly conserved across multiple *Cryptosporidium* species, suggesting a conserved structural feature associated with limited T-cell recognition. Together, these findings demonstrate that strong MHC binding and pMHC stability are insufficient to ensure CD8^+^ T-cell immunogenicity. Instead, the topology of the TCR-accessible peptide surface represents a critical determinant of epitope immunogenicity, with significant implications for epitope selection and vaccine design against cryptosporidiosis.

## Simple Summary

This study provides insight into why the KAV9 epitope from *Cryptosporidium parvum* can bind MHC-I with high affinity but still fails to activate CD8^+^ T cells. Using structural, biochemical, immunological, and computational approaches, we found that KAV9 forms a very stable complex with H-2D^b^, but its peptide surface adopts an unusual topology that may weaken the interactions needed for T-cell receptor (TCR) recognition. Computational modeling further suggests that the lysine at P4 and proline residues at P6 and P8 reshape the surface in ways that may disrupt key interactions with the TCR. As a result, the epitope is poorly recognized by T cells despite its strong MHC binding. These findings reveal a structural basis of weak T-cell recognition and may help guide the design of more effective epitope-based peptide vaccines against cryptosporidiosis.

## 1. Introduction

*Cryptosporidium* spp., obligate apicomplexan parasites, are significant pathogens responsible for severe diarrheal disease in children under five years of age and immunocompromised individuals [1,2]. Among them, *Cryptosporidium parvum* is the primary agent of human infection and represents a substantial public health burden [3]. The Global Enteric Multicenter Study identified it as the second leading cause of severe diarrhea in toddlers, surpassed only by rotavirus [4,5]. Alarmingly, acute infections resulted in over 48,000 deaths among children in 2016 alone [6]. Beyond mortality, even asymptomatic infections can impair nutrient absorption, contributing to chronic malnutrition and growth stunting [4,6,7]. The therapeutic landscape remains bleak: nitazoxanide, the sole FDA-approved drug, is largely ineffective in immunocompromised patients, and no vaccine is currently available [8,9].

Host defense against this intracellular parasite predominantly relies on cell-mediated immunity, particularly CD8^+^ T-cell and CD4^+^ Th1 responses [3,10,11,12,13]. The recognition of intracellular pathogens by CD8^+^ T cells is primarily mediated by the interaction between the T-cell receptor (TCR) and specific parasite-derived peptides presented by major histocompatibility complex class I (MHC-I) molecules [14]. Structural analyses have demonstrated that these peptides bind within the peptide-binding groove (PBG) of MHC-I, forming a trimolecular peptide-MHC complex (pMHC) consisting of the MHC-I heavy chain, the peptide epitope, and β_2_-microglobulin (β_2_m) [15]. These stable pMHC complexes are translocated to the cell surface, where they are recognized by cytotoxic T lymphocytes (CTLs) through specific TCRs, thereby initiating cellular immune responses that facilitate pathogen clearance [16]. However, not all pMHC complexes elicit equivalent T-cell responses, and peptides that stimulate the most abundant and effective T-cell populations are defined as immunodominant epitopes [16,17,18]. Traditionally, most studies have focused on immunodominant epitopes capable of inducing robust T-cell responses [11,19]. Nevertheless, accumulating evidence suggests that *C. parvum* encodes a broad repertoire of peptides that can bind host MHC-I molecules, such as murine H-2D^b^, a class I molecule of the H-2^b^ haplotype that presents pathogen-derived antigenic peptides to CD8^+^ T cells, with high affinity. Notably, despite this strong binding capacity, many of these peptides remain immunologically silent in vivo. The mechanisms underlying this apparent discrepancy between peptide binding and T-cell activation remain poorly understood.

Similar to other apicomplexan parasites, *C. parvum* sporozoites employ specialized secretory organelles to release a complex array of invasion-associated proteins during host cell entry. These proteins facilitate parasite adhesion, gliding motility, and the formation of the parasitophorous vacuole (PV) [20,21]. Among them, Cp23—an approximately 23 kDa surface protein of *C. parvum*—is a major immunodominant antigen implicated in parasite attachment and host cell recognition [9,21]. Recent studies further indicate that Cp23 is predominantly localized at the parasite pellicle and is integral to sporozoite re-invasion of host cells [22]. Sequence analysis reveals that the mature protein comprises 105 amino acids, with a theoretical molecular mass of approximately 11 kDa. However, despite its predicted small mass, Cp23 exhibits an apparent molecular weight of approximately 23 kDa on SDS-PAGE. This significant discrepancy is attributed to potential O-linked glycosylation within its mucin-like domains, as well as anomalous electrophoretic migration due to its high proline content [23]. Notably, the proline content within these 105 amino acids is 17.1%, markedly higher than that of other known pathogenic proteins of *Cryptosporidium*. These dense proline-alanine (PA) repeat motifs may confer significant molecular rigidity to the protein.

In this study, we resolved the crystal structure of the mouse H-2D^b^-KAV9 complex. The *C. parvum* nonapeptide Cp23-KAV9 (KAVKNPAPI; positions 36–44, hereafter referred to as KAV9) was identified as the highest-affinity binder within Cp23. Our structural and immunological analyses reveal a potential immune evasion mechanism associated with Cp23: although the proline residues at positions P6 and P8 significantly enhance the thermal stability of the pMHC complex, their surface-exposed topology may create a steric and/or conformational mismatch that could impair effective TCR engagement. This phenomenon of high pMHC stability coupled with poor TCR recognition provides a structural basis for reduced T-cell activation [3], characterized by continuous antigen stimulation without effective TCR signaling. Consequently, Cp23 may induce a state of functional unresponsiveness in specific T cells through such “defective epitopes”, thereby facilitating immune evasion. Notably, the high conservation of the KAV9 epitope across diverse *Cryptosporidium* species suggests that this mechanism may represent a conserved strategy for evading host immune surveillance.

## 2. Materials and Methods

### 2.1. Peptide Prediction, Selection, and Preparation

Potential mouse MHC-binding peptides derived from the major pathogenic protein Cp23 of *C. parvum* were predicted using the NetMHCpan-4.1 server (https://services.healthtech.dtu.dk/services/NetMHCpan-4.1/, accessed on 29 December 2024) [24]. Among the candidate peptides, Cp23-KAV9 was predicted to exhibit the highest binding affinity for H-2D^b^. For in vitro structural comparisons and thermal stability assays, we selected the well-characterized immunodominant H-2D^b^-restricted epitope gp33 from lymphocytic choriomeningitis virus (LCMV) (KAVYNFATI; hereafter referred to as gp33) as a reference peptide [25], because the structure of its complex with H-2D^b^ is available in the Protein Data Bank (PDB). Importantly, gp33 was employed solely as an in vitro structural and biophysical benchmark and was not included in the in vivo immunization assays because it is an LCMV-derived epitope and is therefore not biologically relevant to the *C. parvum* infection-primed mouse model. For *C. parvum* infection-prime/peptide-boost experiments, we selected a previously identified *C. parvum*-specific immunodominant epitope MEDLE2-INF10 (hereafter INF10) as the positive control [26]. All peptides were synthesized to >90% purity by reverse-phase high-performance liquid chromatography (RP-HPLC) and validated by mass spectrometry (SciLight Biotechnology, Beijing, China). Lyophilized peptides were stored at −80 °C and dissolved in DMSO before use.

### 2.2. Macromolecule Production and In Vitro Refolding Assays

The heavy chain H-2D^b^ and the light chain (mouse β2-microglobulin, mβ2m) were expressed as inclusion bodies, purified, and subsequently assembled into pMHC complexes (H-2D^b^-mβ2m-peptide) through refolding assays, as previously described [26]. Specifically, the H-2D^b^ heavy chain and mβ2m inclusion bodies were individually dissolved in a solution comprising 50 mM Tris-HCl (pH 8.0) and 6 M guanidine-HCl. Then, the H-2D^b^, mβ2m, and peptide were co-refolded by gradual dilution into a refolding buffer (100 mM Tris-HCl, pH 8.0; 2 mM EDTA; 400 mM L-arginine–HCl; 0.5 mM oxidized glutathione; and 5 mM reduced glutathione) at 4 °C. After 8–12 h of incubation, the soluble portions of the complexes were concentrated and purified by size-exclusion chromatography on a Superdex 200 16/600 HiLoad column (GE Healthcare, Stockholm, Sweden), with the pMHC complex eluting at 74–86 mL. Further purification was performed by Resource Q anion-exchange chromatography, where a distinct peak was observed at 9–13 mS/cm, indicating the formation of stable peptide-H-2D^b^ complexes.

### 2.3. Determination of Protein Thermostability Using CD Spectroscopy

Circular dichroism (CD) analyses of the H-2D^b^-peptide complexes were conducted using a CD spectrometer (Chirascan, Applied Photophysics Ltd., Leatherhead, UK) equipped with a thermal controller. Far-UV CD spectra (180–260 nm) were acquired at a protein concentration of 0.2 mg/mL in a 20 mM Tris-HCl buffer (pH 8.0), employing a 1 mm path length cuvette with a spectral resolution of 0.1 nm. Thermal denaturation curves were determined by monitoring the CD signal at 218 nm as the temperature in the water-jacketed cell increased from 25 °C to 80 °C at a linear heating rate of 1 °C/min. The fraction of unfolded protein was calculated from the mean residue ellipticity (θ) according to standard methodologies. The unfolded fraction (%) is expressed as (*θ* − *θ_N_*)/(*θ_U_* − *θ_N_*), where θ_N_ and θ_U_ represent ellipticity values for the native and fully unfolded states [27]. The melting temperature (*T*_m_) was determined by fitting data to the denaturation curves using the Origin 2021 software (version 9.8.0.200, OriginLab Corporation, Northampton, MA, USA).

### 2.4. Immunizations and Intracellular Cytokine Staining

Female C57BL/6 mice (6–8 weeks old; *n* = 8/group) were procured from the Model Animal Research Centre of Nanjing University (Nanjing, China) and maintained under specific-pathogen-free conditions at 25 °C with autoclaved food and water available ad libitum. Mice were randomly assigned to three groups: the KAV9 peptide-immunized group, an adjuvant-only negative control group, and a positive control group immunized with the *C. parvum* MEDLE2-derived INF10 peptide [26]. On day 0, all mice were orally infected with 1 × 10^6^ *C. parvum* oocysts by gavage. Subcutaneous immunizations were administered on days 14 (prime immunization), 24 (first boost), and 34 (second boost). For the peptide-immunized groups, each mouse received 50 µg of the respective peptide dissolved in 200 μL PBS, emulsified with complete Freund’s adjuvant (CFA) for the priming dose or incomplete Freund’s adjuvant (IFA) for the boosters. Mice in the adjuvant-only negative-control group received an equivalent volume of PBS emulsified with the corresponding adjuvant.

Seven days after the final immunization, eight mice from each group were euthanized to isolate splenocytes for intracellular cytokine staining (ICS). Splenocytes were stimulated with 10 µg/mL of the corresponding peptide, together with 1 µg/mL anti-CD28 antibody (clone 37.51) in the presence of Brefeldin A (eBioScience, Thermo Fisher Scientific, San Diego, CA, USA) for 5–6 h at 37 °C under 5% CO_2_. Cells were then stained with Fixable Viability Dye 510 (BD Biosciences, San Jose, CA, USA), followed by surface staining with anti-CD3 (clone 17A2), anti-CD4 (clone GK1.5), and anti-CD8 (clone 53–6.7) antibodies (BioLegend, San Diego, CA, USA). After fixation and permeabilization using BD Cytofix/Cytoperm (BD Biosciences, San Jose, CA, USA), intracellular IFN-γ, TNF-α, and IL-2 were detected using anti-IFN-γ (clone: XMG1.2), anti-TNF-α (clone: MP6-XT22), and anti-IL-2 (clone: JES6-5H4) antibodies. Samples were acquired on a NovoCyte 3000 flow cytometer (Agilent Technologies, Santa Clara, CA, USA), and data were analyzed using FlowJo (v10.8) and GraphPad Prism (v8.0). Gating was performed sequentially on singlets, live CD3^+^ T cells, and CD8^+^ T cell subsets to quantify IFN-γ^+^, TNF-α^+^, and IL-2^+^ populations. All animal handling and experimental procedures adhered to the guidelines outlined in the Guide for the Care and Use of Laboratory Animals by the Ministry of Health, China [28]. The experimental protocol was approved by the Institutional Animal Care and Use Committee of Zhoukou Normal University on 10 December 2022 (authorization number IACUC-Henan-20221210).

### 2.5. Crystallization and Structure Determination

To facilitate crystallization, the purified H-2D^b^-KAV9 complex was concentrated to 5–10 mg/mL in a buffer containing 20 mM Tris (pH 8.0) and 50 mM NaCl. Crystals were grown using the sitting-drop vapor diffusion method at 18 °C by mixing the protein samples with reservoir solutions at a 1:1 ratio. Diffraction-quality crystals were obtained in PEG/Ion 2 screen 47 (1% [*w*/*v*] tryptone, 0.05 M HEPES sodium, pH 7.5, and 12% [*w*/*v*] PEG 3350). X-ray diffraction data were collected at a wavelength of 0.96183 Å using a Pilatus3 6M detector at the Shanghai Synchrotron Radiation Facility (SSRF), China [29]. The collected diffraction images were indexed, integrated, and scaled using HKL3000. The structure was solved by molecular replacement using MOLREP [30], with the native H-2D^b^ structure (PDB: 1FFO) as the initial search model [31]. Subsequent model building and refinement were performed using Coot and Refmac5 (within the CCP4i suite), respectively [32,33]. Structural analysis and figures were generated using PyMOL (version 1.6.x, Schrödinger, LLC, New York, NY, USA).

### 2.6. Structure Prediction Using AlphaFold3

The three-dimensional structures of the H-2D^b^-peptide-P14TCR ternary complexes were predicted using AlphaFold3 (AF3) [34]. The sequences of the P14 TCR α- and β-chains were obtained from the experimentally determined structure of the H-2D^b^-gp33-P14TCR complex (PDB ID: 5TJE), which served as a structural reference [35]. Given that KAV9 (KAVKNPAPI) and gp33 (KAVYNFATM) exhibit differences at critical TCR-contacting residues, specifically at positions P4, P6, and P8, the P14 TCR structure was employed as a comparative benchmark to assess KAV9 and its engineered variants. For each complex, the input sequences comprised H-2D^b^ heavy chain, mβ2m, the P14 TCR α and β chains, and the corresponding peptide (gp33 or KAV9). Predictions were performed using the public AF3 server (https://www.alphafoldserver.com) with default settings, resulting in the generation of five models, each subjected to ten recycling iterations [34]. From each set, the model exhibiting the highest composite ranking score was initially selected. To rigorously evaluate the reliability of the predicted ternary interfaces, specifically the TCR-peptide and TCR-MHC interactions, we assessed key confidence metrics, including the predicted Template Modeling score (pTM), interface pTM (ipTM), ranking score, and the Predicted Aligned Error (PAE). In addition, the interface-predicted Local Distance Difference Test (ipLDDT) score was calculated to estimate local confidence at the pMHC-TCR interface. The PAE matrix was examined to assess residue-pair-specific positional confidence, with lower PAE values indicating greater confidence in the relative positioning of residue pairs.

### 2.7. Data Availability

The crystallographic coordinates and structure factors have been deposited in the Protein Data Bank under the accession number 20XK.

## 3. Results

### 3.1. Analysis of the Binding Affinity and Stability of H-2D^b^-KAV9

To identify potential H-2D^b^-restricted epitopes derived from Cp23, we employed NetMHCpan-4.1 to predict the binding of 8- to 10-mer peptides [24]. Among the candidate peptides, Cp23-KAV9 (KAVKNPAPI) exhibited the highest predicted affinity for H-2D^b^, being classified as a strong binder (SB) with an IC_50_ of 7.83 nM, %Rank_EL of 0.004, and %Rank_BA of 0.007. Notably, these predicted binding metrics were comparable to those of the well-characterized immunodominant LCMV gp33 epitope (Table 1). Collectively, these results suggest that Cp23-KAV9 is a candidate H-2D^b^-restricted epitope for further experimental validation.

The predictions for H-2D^b^ binding were conducted using the NetMHCpan-4.1 server (https://services.healthtech.dtu.dk/services/NetMHCpan-4.1/, accessed on 29 December 2024), which offers predictions of peptide-MHC class I binding and presentation by integrating eluted ligand (EL) and binding affinity (BA) data. The %Rank represents the percentile ranking of the predicted binding affinity relative to a set of random natural peptides; lower %Rank_EL and %Rank_BA values indicate stronger predicted binding and/or presentation. BA predictions assess a peptide’s ability to bind an MHC molecule, while EL predictions also incorporate the likelihood of natural processing and presentation. Predicted binding affinities are reported as IC_50_ values in nanomolar (nM) units, with lower IC_50_ values indicating higher affinity. Peptides were classified as strong binders (SB) according to the default NetMHCpan-4.1 threshold of %Rank_EL < 0.5%.

**Figure 1:**
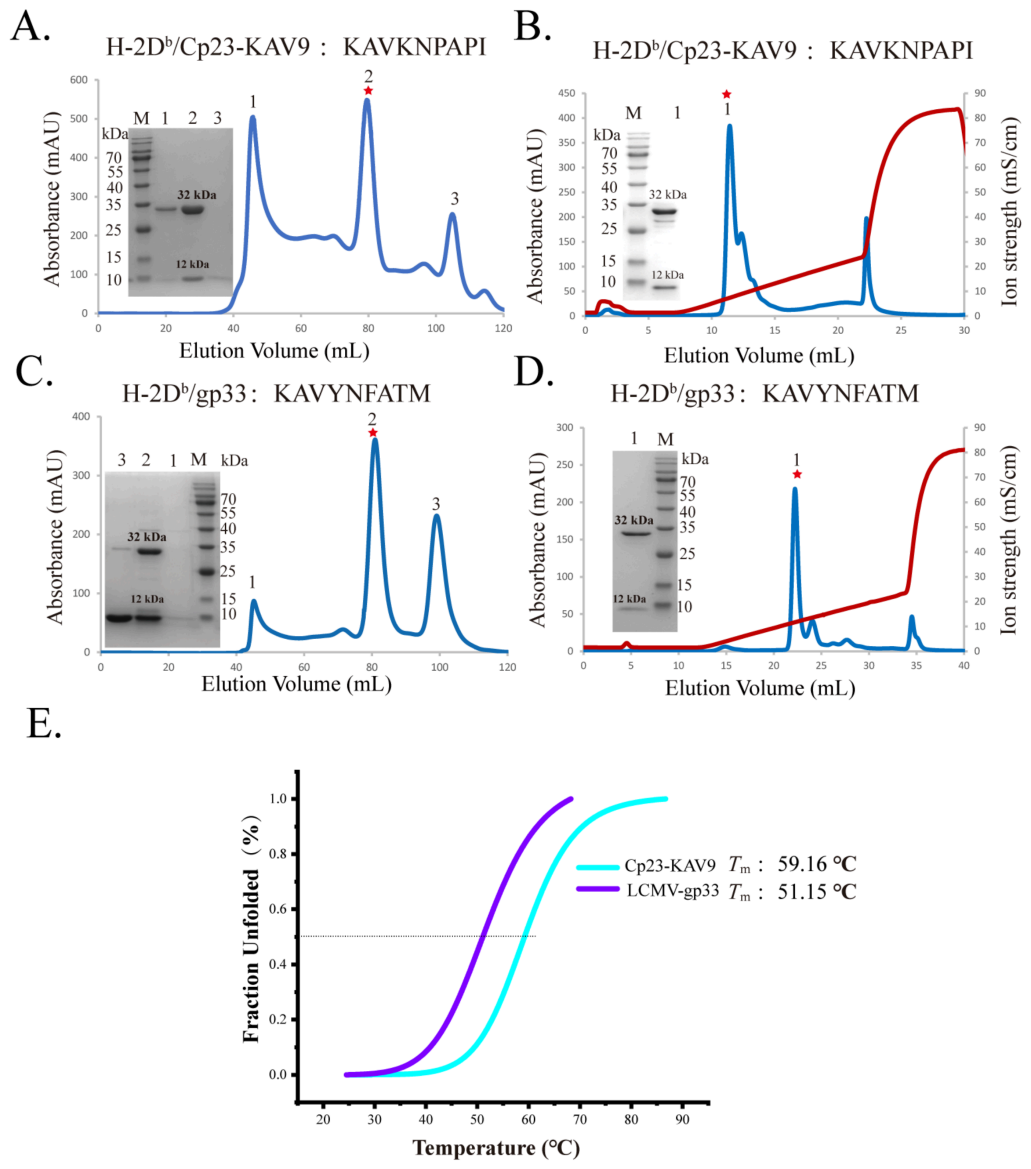
In vitro refolding and thermal stability analysis of the H-2D^b^-KAV9 complex. (**A**) Size-exclusion chromatography (SEC) profile of the refolded H-2D^b^-KAV9 complex. Peak 1 corresponds to the aggregated heavy chain, peak 2 corresponds to the properly assembled H-2D^b^-KAV9 complex, and peak 3 corresponds to excess β2m. Inset: Reducing SDS-PAGE (12%) analysis of fractions from the respective peaks. (**B**) Anion-exchange chromatography (Resource Q) profile of the SEC-purified H-2D^b^-KAV9 complex. The complex eluted as a single sharp peak at approximately 9–13 mS/cm (marked with a red asterisk). Inset: SDS-PAGE analysis confirming the purity of the eluted complex. (**C**) SEC profile of the control H-2D^b^-gp33 complex, showing a similar elution pattern to that of KAV9. Inset: SDS-PAGE analysis of the corresponding peaks. (**D**) Anion-exchange chromatography profile of the H-2D^b^-gp33 complex, demonstrating a purification profile comparable to that of the H-2D^b^-KAV9 complex. (**E**) Thermal denaturation curves monitored by circular dichroism (CD) at 218 nm for H-2D^b^-KAV9 and H-2D^b^-gp33 complexes. The melting temperatures (*T*_m_) of the H-2D^b^-KAV9 and H-2D^b^-gp33 complexes were 59.16 °C and 51.15 °C, respectively. These results demonstrate that KAV9 confers enhanced thermal stability to the H-2D^b^ complex compared to the immunodominant gp33 peptide.

The assembly and stability of the H-2D^b^-KAV9 complex were further evaluated using an in vitro refolding assay and circular dichroism analysis (Figure 1). After refolding, the assembled H-2D^b^-KAV9 pMHC complex was purified by size-exclusion chromatography (SEC), and the target fraction eluting at approximately 74–86 mL was collected for subsequent analysis. SDS-PAGE analysis of the corresponding SEC fractions confirmed that the major elution peak 2 contained the correctly assembled H-2D^b^-KAV9 complex (Figure 1A). Subsequently, the purified H-2D^b^-KAV9 complex was further analyzed by Resource Q anion-exchange chromatography, which revealed a major elution peak at approximately 9–13 mS/cm (Figure 1B). Similarly, the control H-2D^b^-gp33 complex was characterized by both size-exclusion and anion-exchange chromatography, yielding refolding and purification profiles comparable to those of the KAV9 complex (Figure 1C,D). The similar chromatographic profiles of H-2D^b^-KAV9 and H-2D^b^-gp33 complexes suggest that KAV9 can be efficiently refolded and purified as a stable H-2D^b^-peptide complex. Furthermore, the thermal stability of the refolded complexes was assessed through circular dichroism-based thermal denaturation analysis. The melting temperature of the H-2D^b^-KAV9 complex was determined to be 59.16 °C, which was higher than that of the H-2D^b^-gp33 complex (*T*_m_ = 51.15 °C) (Figure 1E), indicating that the H-2D^b^-KAV9 complex exhibits enhanced thermal stability. This elevated *T*_m_ suggests that KAV9 forms a thermally stable complex with H-2D^b^, which may support stable antigen presentation.

### 3.2. Cp23-KAV9 Immunization Fails to Elicit Detectable Peptide-Specific CD8^+^ T-Cell Responses In Vivo

**Figure 2:**
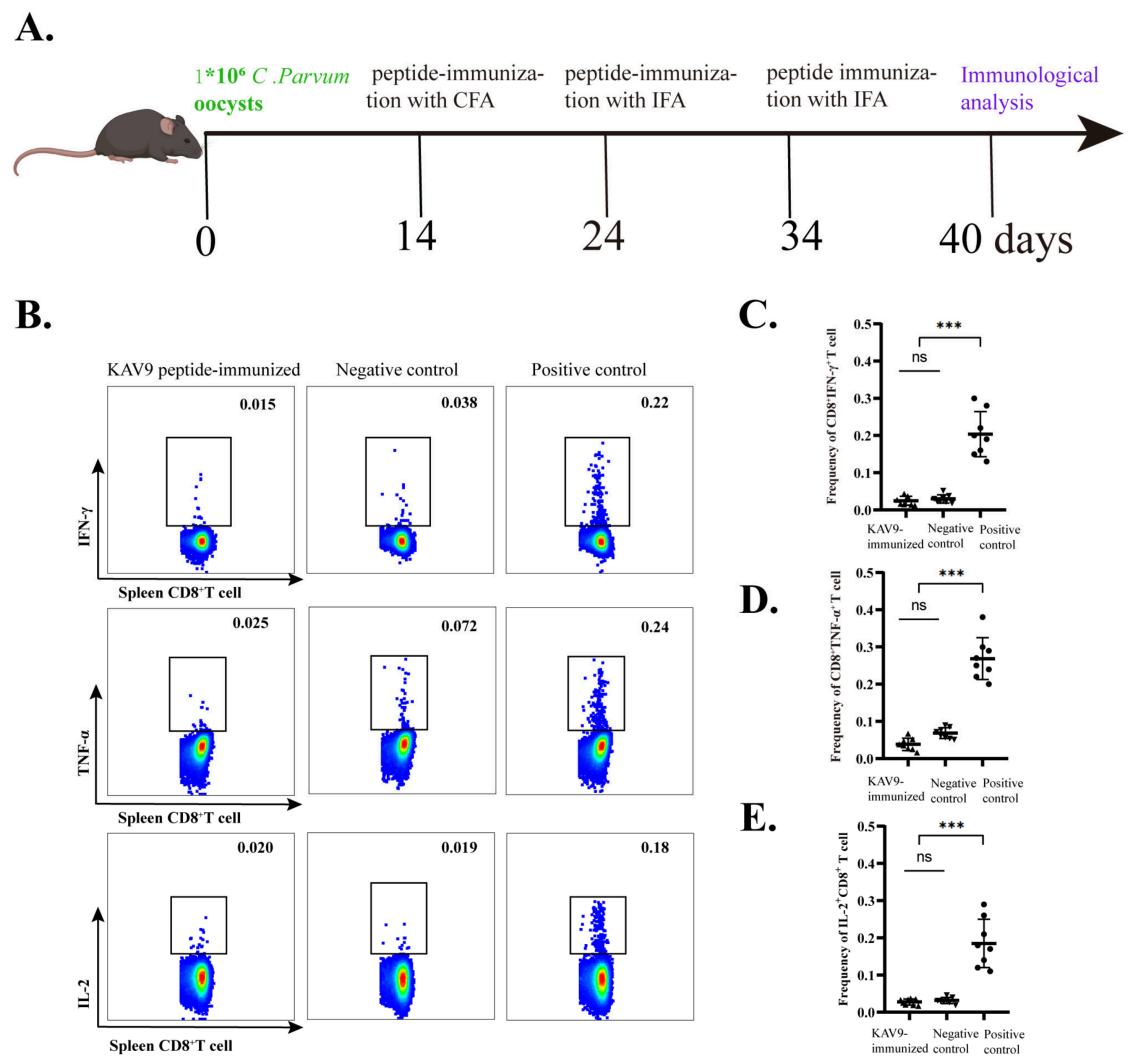
Cp23-KAV9 immunization fails to elicit detectable peptide-specific CD8^+^ T cell responses in vivo. (**A**) Experimental timeline for infection and immunization. C57BL/6 mice were infected with *C. parvum* oocysts (day 0), followed by subcutaneous immunizations on days 14, 24, and 34. Peptides were emulsified in Freund’s Complete Adjuvant (CFA) for the prime and Freund’s Incomplete Adjuvant (IFA) for booster injections. (**B**) Representative flow cytometry gating strategy and flow plots used to identify peptide-specific CD8^+^ T cells. Sequential gating was performed on lymphocytes, single cells, live CD3^+^ T cells, and CD8^+^ T cells to assess intracellular IFN-γ, TNF-α, and IL-2 production. (**C**–**E**) Frequencies of cytokine-producing CD8^+^ T cells following ex vivo peptide restimulation. Splenic CD8^+^ T cells were isolated from immunized mice and restimulated ex vivo with their cognate peptide in the presence of anti-CD28. Frequencies of (**C**) IFN-γ^+^, (**D**) TNF-α^+^, and (**E**) IL-2^+^ CD8^+^ T cells were measured. Immunization with Cp23-KAV9 yielded cytokine responses indistinguishable from the negative control (ns, not significant, *p* > 0.05), whereas the positive control, the immunodominant epitope INF10, elicited significant T cell activation (*** *p* < 0.001). Data are presented as mean ± SD (*n* = 8 per group) and are representative of three independent experiments. Statistical significance was determined using one-way ANOVA with Dunnett’s multiple comparisons test.

Given these favorable biochemical properties observed in vitro, we next sought to determine whether this stable complex could elicit detectable peptide-specific CD8^+^ T cell responses in vivo. To this end, we employed an infection-prime/peptide-boost mouse immunization model, as detailed in the experimental schematic (Figure 2A). Following sequential infection and peptide immunizations, splenocytes were harvested and T-cell response was evaluated by intracellular cytokine staining (ICS). Unexpectedly, despite its strong H-2D^b^-binding affinity and high thermal stability, KAV9 failed to induce a detectable peptide-specific CD8^+^ T cell response. The frequencies of IFN-γ^+^, TNF-α^+^, and IL-2^+^ CD8^+^ T cells in KAV9-immunized mice were negligible and indistinguishable from those of the negative controls (Figure 2B–E). In contrast, the positive control peptide INF10—an immunodominant epitope derived from the *C. parvum* MEDLE2 protein previously identified by our group [26]—elicited a significant peptide-specific CD8^+^ T cell response characterized by the production of multiple cytokines, thereby validating the experimental system. Collectively, these results indicate that although KAV9 forms a thermally stable complex with H-2D^b^, it fails to trigger an antigen-specific CD8^+^ T cell response in vivo, suggesting that stable pMHC formation alone is insufficient to ensure T cell immunogenicity.

### 3.3. Conformational and Interaction Features of Cp23-KAV9 Presented by H-2D^b^

**Figure 3:**
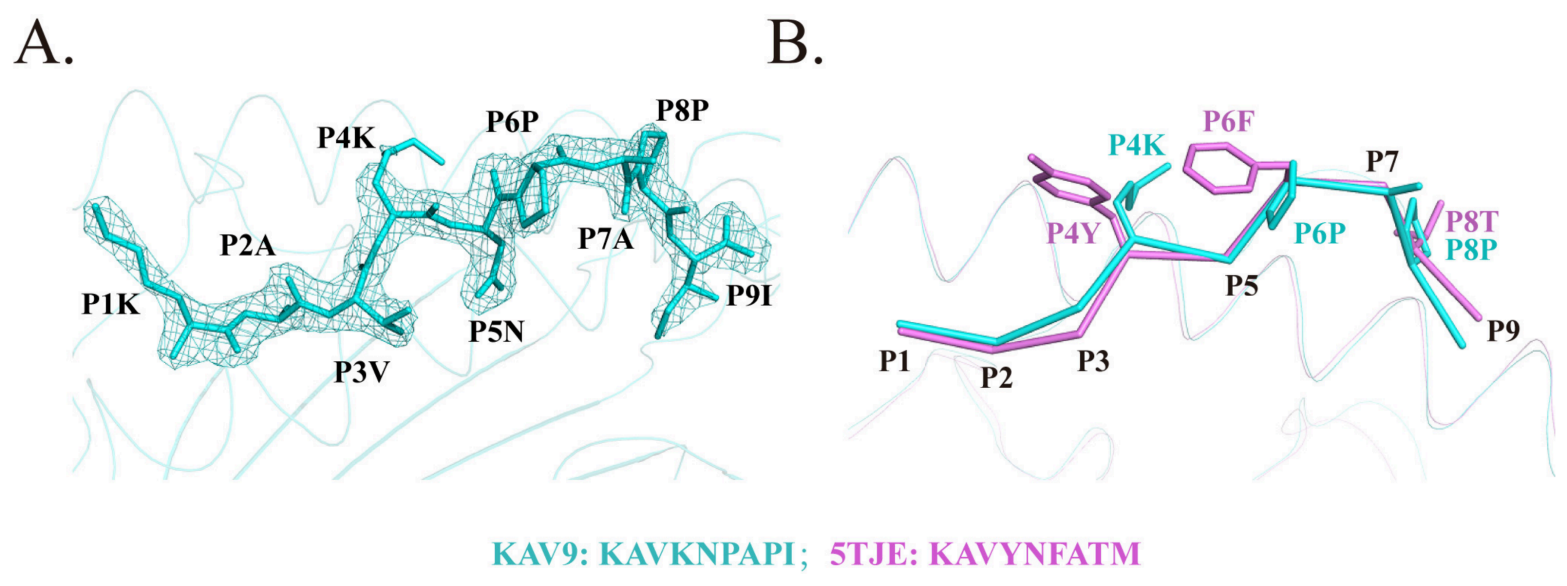
Crystal structure and conformational analysis of the Cp23-KAV9 peptide bound to H-2D^b^. (**A**) Electron density map (2*F_o_* − *F_c_*) contoured at 1σ for the Cp23-KAV9 peptide (KAVKNPAPI), with the peptide depicted as cyan sticks. (**B**) Structural comparison of the peptide backbone conformations between the Cp23-KAV9 peptide (cyan) and the immunodominant LCMV gp33 epitope (KAVYNFATM, PDB: 5TJE, purple). Although the overall main-chain conformations of the two peptides are largely similar, noticeable conformational deviations of individual side chains are observed at positions P4, P6, and P8.

To elucidate the structural basis underlying the limited immunogenicity of Cp23-KAV9, we determined the crystal structure of the H-2D^b^-KAV9 complex. The electron density map of the KAV9 peptide (KAVKNPAPI) was well-defined, providing a reliable foundation for subsequent structural analysis (Figure 3A). Data processing and refinement statistics are summarized in Table 2. Comparative analysis revealed that the amino acid composition and conformation of KAV9 were most similar to those of the immunodominant LCMV gp33 epitope (KAVYNFATM; PDB: 5TJE) [35]. Structural superposition of the H-2D^b^-KAV9 and H-2D^b^-gp33 complexes showed that while the overall backbone conformations of the two peptides were largely similar, noticeable conformational deviations were observed at positions P4, P6, and P8 (Figure 3B).

**Figure 4:**
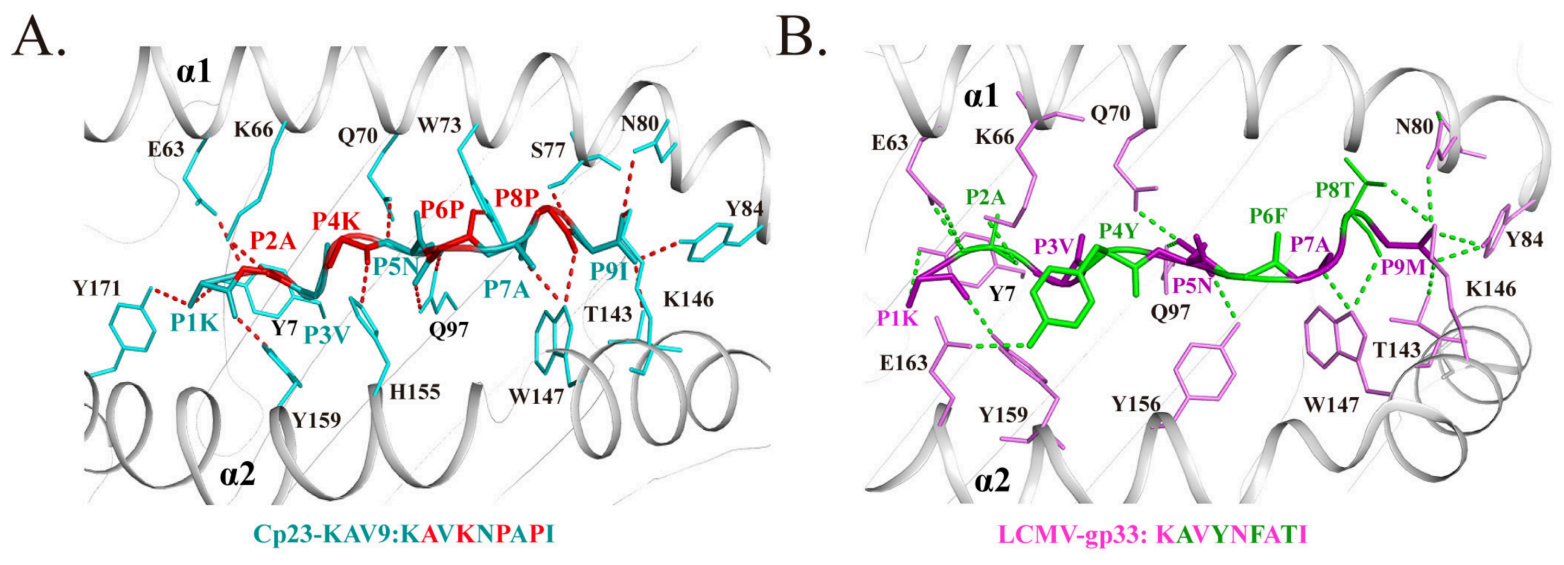
Comparative analysis of intermolecular interaction networks between KAV9 and gp33 peptides within the H-2D^b^ binding groove. (**A**) Detailed hydrogen bond network between the KAV9 peptide and the H-2D^b^ peptide-binding groove. The KAV9 peptide is shown with alternating cyan and red sticks to distinguish individual amino acids. H-2D^b^ residues involved in hydrogen bonding are shown as cyan sticks, and hydrogen bonds are indicated by red dashed lines. (**B**) Hydrogen bond interactions (green dashed lines) between the gp33 peptide and residues in the H-2D^b^ binding groove (shown as purple sticks). The gp33 peptide is shown in alternating purple and green sticks. Both KAV9 and gp33 form 17 hydrogen bonds with H-2D^b^, indicating comparable overall MHC anchoring. However, despite sharing the canonical P5 and P9 anchor positions, the two peptides display distinct local interaction patterns, particularly around P4, P6, and P8, which may contribute to their different TCR-facing surface topologies.

Further investigation into the intermolecular interactions within the H-2D^b^-KAV9 complex revealed that the peptide is firmly anchored within the PBG through an extensive interaction network, consisting of 17 hydrogen bonds, 1 salt bridge, and 430 van der Waals (vdW) contacts (Figure 4A, Table 3). Notably, the immunodominant gp33 peptide forms an identical number of hydrogen bonds with the H-2D^b^ heavy chain (Figure 4B), suggesting that the overall contribution of hydrogen bonding to peptide-MHC binding is comparable between the two complexes. Nevertheless, the specific hydrogen-bonding patterns differ considerably, particularly at residues P4, P6, and P8, where KAV9 and gp33 establish distinct interaction networks with the H-2D^b^ molecule. Specifically, the proline residues at P6 and P8 in KAV9 may impose conformational constraints on the peptide backbone, thereby reducing its flexibility and stabilizing a pre-organized conformation within the binding groove. Although the carbonyl oxygen of P6-Pro forms a hydrogen bond with Trp73, this interaction alone is unlikely to account for the enhanced thermal stability of the H-2D^b^-KAV9 complex. Instead, the increased melting temperature may reflect the combined effects of peptide backbone geometry and favorable van der Waals contacts at key anchor positions, particularly P5 and P9, which display close packing with the H-2D^b^ binding groove (Table 3). Collectively, these structural analyses suggest that pMHC stability is governed not only by the number but also by the spatial arrangement and geometry of interactions; nevertheless, the enhanced thermal stability of an isolated pMHC complex is not, by itself, sufficient to ensure CD8^+^ T-cell immunogenicity.

### 3.4. Structural Insights into H-2D^b^-Restricted Epitope Recognition by P14 TCR

**Figure 5:**
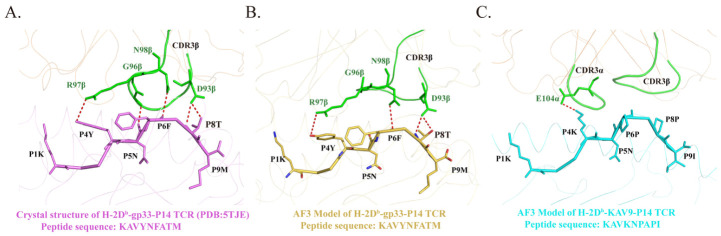
Validation of AF3 prediction reliability and structural analysis of differential TCR engagement between gp33 and KAV9 epitopes. (**A**) Reference hydrogen-bonding interactions between the gp33 peptide (violet) and the CDR3β loop of the P14 TCR (green) in the experimentally determined H-2D^b^-gp33 complex (PDB: 5TJE). Five hydrogen bonds (indicated by red dashed lines) are formed at peptide positions P4 (Y), P6 (F), and P8 (T). (**B**) Detailed view of the AF3 predicted structure for the same complex. The modeled structure accurately reproduces the key interactions, showing the gp33 peptide (gold) forming four hydrogen bonds with the TCR CDR3β loop at the identical peptide positions (P4, P6, and P8). Hydrogen bonds are shown as red dashed lines. (**C**) AF3-predicted interaction network of the H-2D^b^-KAV9-TCR complex. In contrast to the gp33 complex, KAV9 peptide (cyan) exhibits markedly reduced TCR engagement, forming only a single hydrogen bond between peptide residue P4K and E104 of the TCR CDR3α loop, with no detectable hydrogen-bonding interactions involving the CDR3β loop.

In light of the observed structural variations, we propose that the distinct topology of KAV9, particularly in the TCR-facing region, may underlie its inability to elicit a productive CD8^+^ T cell response. To test this hypothesis, we employed AlphaFold3 (AF3) to predict the structures of TCR-pMHC complexes. We first assessed the reliability of this approach by predicting the H-2D^b^-gp33-P14 TCR complex and comparing the resulting model with the experimentally determined crystal structure (PDB: 5TJE). In the reference H-2D^b^-gp33-TCR complex (PDB: 5TJE), the gp33 peptide engages the P14 TCR through a well-defined hydrogen-bonding network involving the CDR3β loop, with five hydrogen bonds formed at peptide positions P4, P6, and P8 (Figure 5A). The AF3 predicted structure (Figure 5B) recapitulated several of the key hydrogen-bond interactions between the TCR and the gp33 peptide, including four hydrogen bonds involving peptide positions P4, P6, and P8. Furthermore, the AF3 model of the H-2D^b^-gp33-P14TCR complex yielded favorable model-confidence metrics (Table 4), including an interface pLDDT score of 95.20, a TCR β-peptide chain-pair iPTM of 0.91, and a minimal interface PAE of 1.36 Å. These values exceed the established thresholds for high-confidence interfaces (I-pLDDT > 87.5, minimal interface PAE ≤ 5.0 Å) [36]. Together with its partial recapitulation of contacts observed in the reference crystal structure, these results support the internal plausibility of the gp33 benchmark model.

Subsequently, we employed this benchmarked computational workflow to predict the structure of the H-2D^b^-KAV9-TCR complex. Unlike the gp33 complex, the predicted H-2D^b^-KAV9-P14 TCR structure exhibited a marked reduction in TCR-peptide interactions. The KAV9 peptide (KAVKNPAPI) forms only a single hydrogen bond with the TCR, specifically between the positively charged P4-Lys (P4K) residue and Glu104 (E104) of the CDR3α loop, instead of the critical P4-R97β interaction present in the gp33 complex (Figure 5C). Notably, no hydrogen-bonding interactions with the CDR3β loop were identified in this model, in contrast to the extensive CDR3β-mediated recognition observed in the gp33 complex. The proline residues at positions P6 and P8 likely preclude the formation of a gp33-like hydrogen-bonding network at these positions, including interactions involving P6F-N98β and P8T-D93β.

Importantly, the apparent loss of a well-defined TCR-peptide interaction network must be interpreted in light of the model’s confidence metrics. Although the predicted complexes retained overall structural similarity to the reference TCR framework (RMSD < 1.2 Å; Table S1), a threshold generally indicative of high-quality structural predictions [37], the specific KAV9-P14 interface exhibited markedly lower confidence scores (Table 4). Specifically, the complex showed an I-pLDDT of 75.52, a notably low TCRβ-peptide chain-pair ipTM of 0.27, and a Minimal Interface PAE of 9.92 Å. These values fail to meet the criteria typically required for reliable interface prediction. Consequently, these metrics do not support the assignment of a stable binding geometry; rather, they suggest that KAV9 may engage P14 TCR in a manner distinct from gp33 or may fail to form a stable gp33-like TCR-peptide interface.

## 4. Discussion

In this study, we integrated structural and immunological methodologies to investigate the molecular basis underlying the poor immunogenicity of the non-immunodominant *C. parvum* epitope Cp23-KAV9. Despite its high affinity for H-2D^b^, this epitope failed to elicit an effective immune response in vivo. Our findings suggest that this lack of immunogenicity cannot be attributed solely to MHC-I binding affinity; instead, it likely arises from structural features of the peptide that create a topological mismatch at the pMHC-TCR interface, thereby impeding productive TCR recognition. Consequently, immunogenicity appears to depend not only on peptide-MHC stability [38] but also on whether the peptide adopts a TCR-facing topology compatible with canonical TCR docking [39].

Based on AF3-modeled structures, our structural analysis suggested that, in H-2D^b^-restricted epitope peptides, hydrogen bond interactions involving key residues at positions P4, P6, and P8 with the TCR CDR3β loop are critical for establishing the topological interface of the pMHC-TCR complex. This observation is consistent with previous studies of the gp33 peptide, in which a P4Y-to-P4F mutation reduced pMHC-TCR affinity by more than 100-fold, highlighting the importance of this interaction, particularly the hydroxyl group of the tyrosine residue, in TCR recognition and binding [40]. Notably, while the computational methodology yielded high-confidence models for gp33, the KAV9-P14 TCR complex consistently produced markedly lower scores across all metrics, including ipTM and interface PAE (Table 4). Given that successful peptide-MHC and pMHC-TCR interface modeling necessitates navigating a complex energy landscape [39,41], it is likely that the P4K residue of the KAV9 peptide introduces structural incompatibilities—such as altered side-chain geometry, local electrostatic repulsion, or the loss of critical TCR anchoring contacts—that disrupt the optimal pMHC-TCR interface [40,42]. Thus, the lower AF3 confidence values should be interpreted not simply as a modeling limitation but as a structural signature of an unfavorable docking landscape for the native peptide. Furthermore, the poor immunogenicity of Cp23-KAV9 may also reflect the conformational constraints imposed by proline residues at positions P6 and P8. Although these residues may contribute to the stabilization of the peptide-MHC complex [35], their inherent rigidity likely restricts the conformational flexibility and accessible surface area required for effective TCR engagement, thereby impeding effective peptide-TCR docking [40,43,44]. Taken together, these findings suggest that T-cell immunogenicity is determined by the interplay between complex stability and the specific docking geometry at the pMHC-TCR interface, where high thermal stability alone is insufficient to compensate for the absence of critical topological features required for TCR recognition [45,46].

The paradoxical nature of the Cp23-KAV9 epitope, characterized by its high affinity for MHC-I yet limited ability to elicit an effective CD8^+^ T-cell response, suggests a disconnect between stable peptide-MHC presentation and effective T-cell activation, which may potentially contribute to immune evasion by *C. parvum*. This structural incompatibility may lead to a TCR-incompatible conformation that diverges from canonical recognition geometries, thereby hindering productive T-cell signaling despite stable peptide presentation. Consequently, KAV9 exemplifies a structurally stable yet immunologically inefficient epitope, in which stable peptide presentation is decoupled from effective T-cell activation. This “high thermodynamic stability, low TCR recognition” profile suggests that KAV9 may function as a “defective epitope”. By persistently occupying MHC-I molecules without triggering productive TCR signaling, such an epitope may facilitate immune evasion by diverting the host immune system toward non-functional antigenic targets. A similar mechanism has been described in SARS-CoV-2, where naturally occurring mutations within an HLA-A24-restricted epitope retained HLA-A24-mediated cell-surface presentation while selectively impairing recognition by epitope-specific CD8^+^ T cells [47]. This precedent supports the notion that KAV9’s immunogenicity is constrained not by inadequate peptide-MHC stability, but by an altered TCR-facing pMHC topology that may not support productive TCR engagement.

To further explore the evolutionary interplay between *Cryptosporidium* and host immunity, we analyzed the sequence conservation of these epitopes across various clinical isolates (Table 5). Notably, the subdominant KAV9 epitope exhibits high conservation at key TCR-contacting positions (P4, P6, and P8) across most zoonotic species within the genus. The complete sequence identity among *C. parvum*, *C. hominis*, *C. tyzzeri*, *C. chipmunk* genotype I, *C. meleagridis*, and *C. ubiquitum*, coupled with the 88% identity in *C. felis* and *C. canis*—while specifically preserving the crucial residues P4-Lys, P6-Pro, and P8-Pro—suggests a significant evolutionary pressure to maintain this non-immunogenic configuration. This remarkable cross-species retention underscores the functional significance of maintaining a structurally stable yet poorly immunogenic epitope and reinforces the possibility that such a configuration is integral to parasite-host immune interactions. However, whether KAV9 influences antigen presentation dynamics or contributes to immune evasion remains to be directly established through experimental investigation.

More broadly, our integrated structural and immunological analysis elucidates why predicted MHC binding alone is insufficient to predict CD8^+^ T-cell immunogenicity [48]. Immunodominance is governed by multiple layers of constraint: anchor residues permit MHC binding, secondary interactions determine pMHC stability [49,50], and peptide conformation shapes the TCR-exposed surface [35,44,51]. Accordingly, accurate prediction of T-cell immunogenicity requires simultaneous consideration of peptide affinity, complex stability, and TCR accessibility at the antigenic interface.

## 5. Limitations

Several limitations in this study warrant acknowledgment. First, although AlphaFold3 predicted that the unique spatial conformation of KAV9 may hinder T-cell receptor (TCR) engagement, this interaction model remains computational in nature. Direct experimental elucidation of the KAV9-H-2D^b^-TCR ternary complex structure, through methods such as cryo-electron microscopy (cryo-EM) or X-ray crystallography, is necessary to empirically validate the proposed topological mismatch at the TCR-facing interface. Second, while our intracellular cytokine staining (ICS) assays demonstrated that KAV9 fails to effectively activate CD8^+^ T cells, we did not assess the direct biophysical interaction between the TCR and the pMHC complex in vitro. Future investigations employing techniques such as surface plasmon resonance (SPR) to quantify the binding affinity and kinetics between the pMHC complex and cognate TCRs would provide deeper mechanistic insights into this potential immune evasion strategy.

## 6. Conclusions

In summary, our study identifies Cp23-KAV9 as a high-affinity H-2D^b^-binding epitope derived from *C. parvum* that nonetheless fails to elicit a detectable CD8^+^ T-cell response in vivo. Structural and biophysical analyses demonstrate that KAV9 forms a highly stable pMHC complex, with stability surpassing that of the canonical H-2D^b^-restricted gp33 reference epitope. However, crystallographic analysis of the H-2D^b^-KAV9 complex, together with AlphaFold3-based modeling, suggests that KAV9 adopts a distinct peptide topology, particularly at positions P4, P6, and P8, which may limit productive TCR engagement. These findings support the characterization of KAV9 as a “structurally stable yet immunologically silent” epitope, indicating that stable MHC binding alone is insufficient to ensure T-cell immunogenicity and that the TCR-facing topology of the presented peptide may be a critical determinant of epitope recognition. More broadly, these findings underscore the limitations of affinity-based epitope prediction and highlight structural features that may inform future epitope prioritization and vaccine-design strategies against cryptosporidiosis.

## Figures and Tables

**Table 1:** NetMHCpan-4.1-predicted H-2D^b^-binding metrics for Cp23-KAV9 and the immunodominant LCMV gp33 epitope.

Peptide Name	Sequence	%Rank_EL	%Rank_BA	Aff (nM)	Bind Level
Cp23-KAV9	KAVKNPAPI	0.004	0.007	7.83	SB
LCMV gp33	KAVYNFATI	0.004	0.006	5.97	SB

**Table 2:** Data collection and refinement statistics for X-ray diffraction.

Parameter	H-2D^b^-KAV9 *^a^*
Data processing statistics	
space group	C121
Unit-cell parameters	
*a*, *b*, *c* (Å)	91.70, 111.11, 58.04
*α*, *β*, *γ* (^o^)	90.00, 122.25, 90.00
Resolution (Å)	43.88–2.10 (2.20–2.27)
No. of reflections	168,328 (14,268)
Unique reflections	24,959 (2179)
Completeness (%)	99.9 (100)
Average I/σ (I)	15.3 (2.1)
*R*_merge_ (%) *^b^*	4.7 (59.4)
CC_1/2_ (%) *^c^*	0.999 (0.854)
Refinement	
*R*_work_ (%) *^d^*	22.74
*R*_free_ (%)	27.54
RMSD from ideality *^e^*	
Bond lengths (Å)	0.007
Bond angles (°)	1.134
Average B factor	57.846
Ramachandran plot quality	
Most favored (%)	93.05
Allowed region (%)	6.68
Disallowed (%)	0.27

*^a^* Values in parentheses are for the highest-resolution shell. *^b^ R*_merge_ = Σ*_hkl_* Σ_i_|*I*_i_(*hkl*) <*I*(*hkl*)> |/Σ*_hkl_* Σ_i_ *I*_i_(*hkl*), where *I*_i_ (*hkl*) is the observed intensity and <*I*(*hkl*)> is the average intensity from multiple measurements. *^c^* CC1/2, percentage of correlation between intensities from random half-datasets. *^d^* R = Σ*_hkl_* ||F*_obs_*| − k|F*_calc_* ||Σ *_hkl_* |F*_obs_*|, where R_free_ is calculated for a randomly chosen 5% of reflections and R_work_ is calculated for the remaining 95% of reflections used for structure refinement. *^e^* RMSD, root mean square deviation.

**Table 3:** Hydrogen bonds and van der Waals contacts between the peptide and the PBG in the H-2D^b^-KAV9 complex.

Peptide		Hydrogen Bond Partner		van der Waals Contact Residues ^a^
Residue	Atom	Residue	Atom	
P1-Lys	N	Tyr7	OH	Met5, Tyr7, Tyr59, Glu63, Lys66, Tyr159, Glu163, Trp167, Tyr171(94)
	N	Tyr171	OH	
	O	Tyr159	OH	
P2-Ala	N	Glu63	OE1	Tyr7, Tyr45, Glu63, Lys66, Tyr159, Glu163 (47)
	O	Lys66	NZ	
P3-Val				Glu9, Lys66, Gln70, Gln97, Ser99, Leu114, Tyr159 (37)
P4-Lys	O	His155	NE2	Lys66, Gly69, Gln70, His155 (20)
P5-Asn	N	Gln70	OE1	Gln70, Trp73, Phe74, Gln97, Phe116, His155, Tyr156 (73)
	ND2	Gln97	OE1	
	OD1	Gln97	NE2	
P6-Pro	O	Trp73	NE1	Trp73, Ser150, Gly151, Ala152, His155, Tyr156 (32)
P7-Ala	O	Trp147	NE1	Trp73, Trp147, Ser150 (21)
P8-Pro	O	Trp147	NE1	Trp73, Val76, Ser77, Lys146, Trp147 (33)
P9-Ile	N	Ser77	OG	Trp73, Ser77, Asn80, Leu81, Tyr84, Thr143, Lys146, Trp147 (73)
	O	Asn80	ND2	
	O	Lys146	NZ	
	OXT	Tyr84	OH	
	OXT	Thr143	OG1	
	O	Lys146	NZ	Salt bridges

^a^ Numbers in parentheses indicate the total number of van der Waals contacts. Hydrogen bond (cut-off distance 3.5 Å). Salt bridge (cut-off distance 4 Å). Van der Waals (cut-off distance 4.5 Å).

**Table 4:** AlphaFold3 confidence metrics and structural validation data for the predicted complexes.

Name of Complex	Ranking Score	pTM	ipTM	I-pLDDT	Chain_Pair_iPTM	Minimal Interface PAE (Å)
H-2D^b^-gp33-P14TCR	0.89	0.9	0.89	95.20	TCR α-peptide: 0.85 TCR β-peptide: 0.91 H-2D^b^-TCR α: 0.91 H-2D^b^-TCR β: 0.84	TCR α-peptide: 1.28 TCR β-peptide: 1.36 H-2D^b^-TCR α: 1.12 H-2D^b^-TCR β: 1.31
H-2D^b^-KAV9-P14TCR	0.56	0.62	0.53	75.52	TCR α-peptide: 0.24 TCR β-peptide: 0.27 H-2D^b^-TCR α: 0.23 H-2D^b^-TCR β: 0.28	TCR α-peptide: 10.12 TCR β-peptide: 9.92 H-2D^b^-TCR α: 14.36 H-2D^b^-TCR β: 9.67

Note: For each complex, the model with the lowest root-mean-square deviation (RMSD) of the TCR compared to the reference structure (PDB: 5TJE) was selected for analysis (detailed RMSD values and model rankings are provided in Table S1). Interpretations of key confidence scores are as follows. Ranking score: Composite confidence score used to rank multiple models (higher values indicate overall better prediction quality). pTM (predicted Template Modeling score): A global confidence metric (0–1) for the overall model accuracy of the complex. ipTM (interface predicted Template Modeling score): Confidence metric (0–1) specifically for the accuracy of predicted interfaces between chains. Scores > 0.8 indicate high-confidence interfaces; scores < 0.6 suggest likely incorrect interfaces. I-pLDDT (Interface pLDDT): The average pLDDT score of all residues at the TCR-pMHC interface, calculated as the mean pLDDT of residues with any atom within 4 Å of the binding partner. I-pLDDT > 87.5 is associated with high-accuracy TCR-pMHC models. Chain pair iPTM: Interface-specific iPTM values for key biological interfaces: TCR α-peptide and TCR β-peptide: confidence for TCR-peptide recognition. H-2D^b^-TCR α and H-2D^b^-TCR β: confidence for MHC-TCR docking. Minimal Interface PAE (Å): The minimum predicted aligned error for each corresponding interface (lower values indicate higher local precision). For MHC-peptide-TCR complexes, an interface prediction is generally considered high-confidence when Chain pair iPTM ≥ 0.70 and Minimal Interface PAE ≤ 5.0 Å.

**Table 5:** Sequence conservation analysis of the Cp23-derived KAV9 epitope across *Cryptosporidium* species.

*Cryptosporidium* spp.	Cp23-KAV9 Homologue	Sequence Identities (%)
*C. parvum*	KAVKNPAPI	100
*C. hominis*	KAVKNPAPI	100
*C. tyzzeri*	KAVKNPAPI	100
*C. chipmunk* genotype I	KAVKNPAPI	100
*C. meleagridis*	KAVKNPAPI	100
*C. ubiquitum*	KAVKNPAPI	100
*C. felis*	KAVKNP**P**PI	88
*C. canis*	KAVKNP**T**PI	88

Note: This table summarizes the amino acid sequence alignment of the identified epitopes across various zoonotic and anthroponotic *Cryptosporidium* species. The reference sequences for Cp23 were obtained from the NCBI database and CryptoDB (https://cryptodb.org/). The NCBI accession numbers are as follows: AEJ22864.1 (*C. hominis*), AAN31184.1 (*C. parvum*), KAF7456923.1 (*C. felis*), XP_067199247.1 (*C. chipmunk* genotype I), KAJ1605854.1 (*C. canis*), XP_028873761.1 (*C. ubiquitum*). The gene IDs from CryptoDB are CTYZ_00002129 (*C. tyzzeri*) and CmeUKMEL1_08080 (*C. meleagridis*). The mutated amino acids are indicated in bold.

## Data Availability

No new data were created or analyzed in this study.

## Supplementary Materials

The following supporting information can be downloaded at https://www.mdpi.com/article/10.3390/biology15151281/s1. Table S1: AlphaFold3 prediction metrics and structural evaluation for P14 TCR complexes.

## Abbreviations

The following abbreviations are used in this manuscript:

MHC-I	Major Histocompatibility Complex Class I
TCR	T-cell Receptor
pMHC	Peptide–MHC Complex
β_2_m	β_2_-microglobulin
CTL	Cytotoxic T Lymphocyte
LCMV	Lymphocytic Choriomeningitis Virus
DMSO	Dimethyl Sulfoxide
CD	Circular Dichroism
*T*_m_	Melting Temperature
CFA	Complete Freund’s Adjuvant
IFA	Incomplete Freund’s Adjuvant
ICS	Intracellular Cytokine Staining
AF3	AlphaFold3
pTM	predicted Template Modeling Score
ipTM	Interface pTM
PAE	Predicted Aligned Error
I-pLDDT	Interface Predicted Local Distance Difference Test
vdW	Van der Waals
RMSD	Root-mean-square Deviation
SPR	Surface Plasmon Resonance

## References

[1] Checkley W., White A.C., Jaganath D., Arrowood M.J., Chalmers R.M., Chen X.M., Fayer R., Griffiths J.K., Guerrant R.L., Hedstrom L. (2015). A review of the global burden, novel diagnostics, therapeutics, and vaccine targets for *Cryptosporidium*. Lancet Infect. Dis..

[2] Casemore D.P., Sands R.L., Curry A. (1985). *Cryptosporidium* species a “new” human pathogen. J. Clin. Pathol..

[3] Pardy R.D., Wallbank B.A., Striepen B., Hunter C.A. (2024). Immunity to *Cryptosporidium*: Insights into principles of enteric re sponses to infection. Nat. Rev. Immunol..

[4] Kotloff K.L., Nasrin D., Blackwelder W.C., Wu Y., Farag T., Panchalingham S., Sow S.O., Sur D., Zaidi A.K.M., Faruque A.S.G. (2019). The incidence, aetiology, and adverse clinical consequences of less severe diarrhoeal episodes among infants and children residing in low-income and middle-income countries: A 12-month case-control study as a follow-on to the Global Enteric Multicenter Study (GEMS). Lancet Glob. Health.

[5] Maradana M.R., Marzook N.B., Diaz O.E., Mkandawire T., Diny N.L., Li Y., Liebert A., Shah K., Tolaini M., Kvac M. (2023). Dietary environmental factors shape the immune defense against *Cryptosporidium* infection. Cell Host Microbe.

[6] Khalil I.A., Troeger C., Rao P.C., Blacker B.F., Brown A., Brewer T.G., Colombara D.V., De Hostos E.L., Engmann C., Guerrant R.L. (2018). Morbidity, mortality, and long-term consequences associated with diarrhoea from *Cryptosporidium* infection in children younger than 5 years: A meta-analyses study. Lancet Glob. Health.

[7] Thomson S., Hamilton C.A., Hope J.C., Katzer F., Mabbott N.A., Morrison L.J., Innes E.A. (2017). Bovine cryptosporidiosis: Impact, host-parasite interaction and control strategies. Vet. Res..

[8] Amadi B., Mwiya M., Sianongo S., Payne L., Watuka A., Katubulushi M., Kelly P. (2009). High dose prolonged treatment with nitazoxanide is not effective for cryptosporidiosis in HIV positive Zambian children: A randomised controlled trial. BMC Infect. Dis..

[9] Mead J.R. (2014). Prospects for immunotherapy and vaccines against *Cryptosporidium*. Hum. Vaccines Immunother..

[10] Acosta Rodriguez E.V., Araujo Furlan C.L., Fiocca Vernengo F., Montes C.L., Gruppi A. (2019). Understanding CD8^+^ T cell immunity to *Trypanosoma cruzi* and how to improve it. Trends Parasitol..

[11] Haskins B.E., Gullicksrud J.A., Wallbank B.A., Dumaine J.E., Guerin A., Cohn I.S., O’Dea K.M., Pardy R.D., Merolle M.I., Shallberg L.A. (2024). Dendritic cell-mediated responses to secreted *Cryptosporidium* effectors promote parasite-specific CD8^+^ T cell responses. Mucosal Immunol..

[12] Cohn I.S., Wallbank B.A., Haskins B.E., O’Dea K.M., Pardy R.D., Shaw S., Merolle M.I., Gullicksrud J.A., Christian D.A., Striepen B. (2024). Intestinal cDC1s provide cues required for CD4^+^ T cell-mediated resistance to *Cryptosporidium*. J. Exp. Med..

[13] Pantenburg B., Castellanos-Gonzalez A., Dann S.M., Connelly R.L., Lewis D.E., Ward H.D., White A.C. (2010). Human CD8^+^ T cells clear *Cryptosporidium parvum* from infected intestinal epithelial cells. Am. J. Trop. Med. Hyg..

[14] Blum J.S., Wearsch P.A., Cresswell P. (2013). Pathways of antigen processing. Annu. Rev. Immunol..

[15] Madden D.R. (1995). The three-dimensional structure of peptide-MHC complexes. Annu. Rev. Immunol..

[16] Macdonald I.K., Harkiolaki M., Hunt L., Connelley T., Carroll A.V., MacHugh N.D., Graham S.P., Jones E.Y., Morrison W.I., Flower D.R. (2010). MHC class I bound to an immunodominant *Theileria parva* epitope demonstrates unconventional presentation to T cell receptors. PLoS Pathog..

[17] Chen W., McCluskey J. (2006). Immunodominance and immunodomination: Critical factors in developing effective CD8^+^ T-cell-based cancer vaccines. Adv. Cancer Res..

[18] Garstka M.A., Fish A., Celie P.H., Joosten R.P., Janssen G.M., Berlin I., Hoppes R., Stadnik M., Janssen L., Ovaa H. (2015). The first step of peptide selection in antigen presentation by MHC class I molecules. Proc. Natl. Acad. Sci. USA.

[19] Wang Y., Gao M., Li X., Zhu W., Zhao M., Li J., Liu X., Cao L., Li S., Zhang S. (2023). Structural analyses of a dominant *Cryptosporidium parvum* epitope presented by H-2K(b) offer new options to combat cryptosporidiosis. mBio.

[20] Smith H.V., Nichols R.A., Grimason A.M. (2005). *Cryptosporidium* excystation and invasion: Getting to the guts of the matter. Trends Parasitol..

[21] Guerin A., Strelau K.M., Barylyuk K., Wallbank B.A., Berry L., Crook O.M., Lilley K.S., Waller R.F., Striepen B. (2023). *Cryptos poridium* uses multiple distinct secretory organelles to interact with and modify its host cell. Cell Host Microbe.

[22] Watson L.C., Sala K.A., Bernitz N., Baumgartel L., Pallett M.A., Marzook N.B., Straker L.C., Peng D., Collinson L., Sateriale A. (2025). Targeted CRISPR screens reveal genes essential for *Cryptosporidium* survival in the host intestine. Nat. Commun..

[23] Bonafonte M.T., Smith L.M., Mead J.R. (2000). A 23-kDa recombinant antigen of *Cryptosporidium parvum* induces a cellular immune response on in vitro stimulated spleen and mesenteric lymph node cells from infected mice. Exp. Parasitol..

[24] Reynisson B., Alvarez B., Paul S., Peters B., Nielsen M. (2020). NetMHCpan-4.1 and NetMHCIIpan-4.0: Improved predictions of MHC antigen presentation by concurrent motif deconvolution and integration of MS MHC eluted ligand data. Nucleic Acids Res..

[25] Masopust D., Murali-Krishna K., Ahmed R. (2007). Quantitating the magnitude of the lymphocytic choriomeningitis virus-specific CD8 T-cell response: It is even bigger than we thought. J. Virol..

[26] Wang Y., Chang Y., Yin F., Kang C., Meng Y., Xu F., Liu Y., Zhang Y., Wu C., Fan S. (2025). Structural analyses of *Cryptosporidium parvum* epitopes reveal a novel scheme of decapeptide binding to H-2K^b^. J. Struct. Biol..

[27] Tobita T., Oda M., Morii H., Kuroda M., Yoshino A., Azuma T., Kozono H. (2003). A role for the P1 anchor residue in the thermal stability of MHC class II molecule I-Ab. Immunol. Lett..

[28] Ogden B.E., Pang William W., Agui T., Lee B.H. (2016). Laboratory Animal Laws, Regulations, Guidelines and Standards in China Mainland, Japan, and Korea. ILAR J..

[29] Xiao Q., Wu T., Bao K., Tang J., Zhang Y., Zhang W., Zhu Z., Gu Y., Zhou S., Li C. (2024). Upgrade of crystallography beamline BL19U1 at the Shanghai Synchrotron Radiation Facility. J. Appl. Crystallogr..

[30] Vagin A., Teplyakov A. (2010). Molecular replacement with MOLREP. Acta Crystallogr. D Biol. Crystallogr..

[31] Wang B., Sharma A., Maile R., Saad M., Collins E.J., Frelinger J.A. (2002). Peptidic termini play a significant role in TCR recognition. J. Immunol..

[32] Emsley P., Lohkamp B., Scott W.G., Cowtan K. (2010). Features and development of Coot. Acta Crystallogr. D Biol. Crystallogr..

[33] Murshudov G.N., Skubak P., Lebedev A.A., Pannu N.S., Steiner R.A., Nicholls R.A., Winn M.D., Long F., Vagin A.A. (2011). REFMAC5 for the refinement of macromolecular crystal structures. Acta Crystallogr. D Biol. Crystallogr..

[34] Abramson J., Adler J., Dunger J., Evans R., Green T., Pritzel A., Ronneberger O., Willmore L., Ballard A.J., Bambrick J. (2024). Accurate structure prediction of biomolecular interactions with AlphaFold 3. Nature.

[35] Duru A.D., Sun R., Allerbring E.B., Chadderton J., Kadri N., Han X., Peqini K., Uchtenhagen H., Madhurantakam C., Pellegrino S. (2020). Tuning antiviral CD8 T-cell response via proline-altered peptide ligand vaccination. PLoS Pathog..

[36] Wu D., Yin R., Chen G., Ribeiro-Filho H.V., Cheung M., Robbins P.F., Mariuzza R.A., Pierce B.G. (2024). Structural characteriza tion and AlphaFold modeling of human T cell receptor recognition of NRAS cancer neoantigens. Sci. Adv..

[37] Shi Y., Parks J.M., Smith J.C. (2025). Comparative analysis of TCR and TCR-pMHC complex structure prediction tools. J. Chem. Inf. Model..

[38] van der Burg S.H., Visseren M.J., Brandt R.M., Kast W.M., Melief C.J. (1996). Immunogenicity of peptides bound to MHC class I molecules depends on the MHC-peptide complex stability. J. Immunol..

[39] Borg N.A., Ely L.K., Beddoe T., Macdonald W.A., Reid H.H., Clements C.S., Purcell A.W., Kjer-Nielsen L., Miles J.J., Burrows S.R. (2005). The CDR3 regions of an immunodominant T cell receptor dictate the ‘energetic landscape’ of peptide-MHC recognition. Nat. Immunol..

[40] Tissot A.C., Ciatto C., Mittl P.R., Grutter M.G., Pluckthun A. (2000). Viral escape at the molecular level explained by quantitative T-cell receptor/peptide/MHC interactions and the crystal structure of a peptide/MHC complex. J. Mol. Biol..

[41] Collesano L., Luksza M., Lassig M. (2024). Energy landscapes of peptide-MHC binding. PLoS Comput. Biol..

[42] Velloso L.M., Michaelsson J., Ljunggren H.G., Schneider G., Achour A. (2004). Determination of structural principles underlying three different modes of lymphocytic choriomeningitis virus escape from CTL recognition. J. Immunol..

[43] Valkenburg S.A., Gras S., Guillonneau C., Hatton L.A., Bird N.A., Twist K.A., Halim H., Jackson D.C., Purcell A.W., Turner S.J. (2013). Preemptive priming readily overcomes structure-based mechanisms of virus escape. Proc. Natl. Acad. Sci. USA.

[44] Turner S.J., Kedzierska K., Komodromou H., La Gruta N.L., Dunstone M.A., Webb A.I., Webby R., Walden H., Xie W., McCluskey J. (2005). Lack of prominent peptide-major histocompatibility complex features limits repertoire diversity in virus-specific CD8^+^ T cell populations. Nat. Immunol..

[45] Harndahl M., Rasmussen M., Roder G., Dalgaard Pedersen I., Sorensen M., Nielsen M., Buus S. (2012). Peptide-MHC class I sta bility is a better predictor than peptide affinity of CTL immunogenicity. Eur. J. Immunol..

[46] Allerbring E.B., Duru A.D., Uchtenhagen H., Madhurantakam C., Tomek M.B., Grimm S., Mazumdar P.A., Friemann R., Uhlin M., Sandalova T. (2012). Unexpected T-cell recognition of an altered peptide ligand is driven by reversed thermodynamics. Eur. J. Immunol..

[47] Zornikova K., Dianov D., Ivanova N., Davydova V., Nenasheva T., Fefelova E., Bogolyubova A. (2024). Features of Highly Homologous T-Cell Receptor Repertoire in the Immune Response to Mutations in Immunogenic Epitopes. Int. J. Mol. Sci..

[48] Calis J.J., Maybeno M., Greenbaum J.A., Weiskopf D., De Silva A.D., Sette A., Kesmir C., Peters B. (2013). Properties of MHC class I presented peptides that enhance immunogenicity. PLoS Comput. Biol..

[49] Busch D.H., Pamer E.G. (1998). MHC class I/peptide stability: Implications for immunodominance, in vitro proliferation, and diversity of responding CTL. J. Immunol..

[50] Lazarski C.A., Chaves F.A., Jenks S.A., Wu S., Richards K.A., Weaver J.M., Sant A.J. (2005). The kinetic stability of MHC class II: Peptide complexes is a key parameter that dictates immunodominance. Immunity.

[51] Miley M.J., Messaoudi I., Metzner B.M., Wu Y., Nikolich-Zugich J., Fremont D.H. (2004). Structural basis for the restoration of TCR recognition of an MHC allelic variant by peptide secondary anchor substitution. J. Exp. Med..

