# Supplementary material for: Structural Basis for the Immunological Paradox of a High-Affinity Yet Non-Immunogenic MHC-I Epitope from Cryptosporidium parvum
Source: Biology (Basel). 2026 Aug 4;15(15):1281. doi: 10.3390/biology15151281 (PMC13465150; doi:10.3390/biology15151281)
Supplement: Supplementary file 1 [file biology-15-01281-s001.zip › biology-4402755-supplementary.pdf]

Table S1. AlphaFold3 prediction metrics and structural evaluation for P14 TCR complexes.

Complex	AF3 Model	Ranking Score [a]	TCR RMSD to the crystal structure (PDB: 5TJE) (Å) [b]	Docking Topology [c]	Selection Status
H-2D^b-gp33-P14 TCR (Validation Control)	Model 0	0.9	1.687	Canonical docking	Not selected
	Model 1	0.89	0.764	Canonical docking	Selected
	Model 2	0.89	2.036	Canonical docking	Not selected
	Model 3	0.89	1.550	Canonical docking	Not selected
	Model 4	0.89	1.306	Canonical docking	Not selected
H-2D^b-KAV9-P14 TCR	Model 0	0.56	1.199	Canonical docking	Selected
	Model 1	0.56	1.417	Canonical docking	Not selected
	Model 2	0.55	-	Non-canonical (TCR reversal)	Unusable
	Model 3	0.54	-	Non-canonical (TCR reversal)	Unusable
	Model 4	0.54	-	Non-canonical (TCR reversal)	Unusable

[a] Ranking Score represents the model confidence generated by AlphaFold3.
[b] TCR RMSD values quantify the spatial displacement of the predicted TCR. The predicted complexes were first superimposed onto the crystal reference (PDB: 5TJE), and the RMSD was subsequently calculated exclusively for the TCR α and β chains.
[c] For the H-2D^b-KAV9-P14 TCR predictions, Models 2, 3, and 4 exhibited a completely non-canonical docking topology, wherein the TCR α and β chains were docked in an inverted orientation relative to the peptide-MHC surface. Because this reverse-polarity binding is physiologically unviable, coordinate-based RMSD calculations for the TCR are not biologically meaningful and are thus omitted (denoted by "-").
